# Socioeconomic position, stage of lung cancer and time between referral and diagnosis in Denmark, 2001–2008

**DOI:** 10.1038/bjc.2011.342

**Published:** 2011-09-06

**Authors:** S O Dalton, B L Frederiksen, E Jacobsen, M Steding-Jessen, K Østerlind, J Schüz, M Osler, C Johansen

**Affiliations:** 1 Department of Psychosocial Cancer Research, Institute of Cancer Epidemiology, Danish Cancer Society, 49 Strandboulevarden, 2100 Copenhagen, Denmark; 2 Research Centre for Prevention and Health, Glostrup University Hospital, 2600 Glostrup, Denmark; 3 The Danish Lung Cancer Registry, Department of Thoracic Surgery, Odense University Hospital, 5000 Odense, Denmark; 4 Department of Statistics and Epidemiology, Institute of Cancer Epidemiology, Danish Cancer Society, 2100 Copenhagen, Denmark; 5 Department of Oncology, Copenhagen University Hospital, 2200 Copenhagen, Denmark; 6 Section of Environment and Radiation, International Agency for Research on Cancer (IARC), 69372 Lyon, France

**Keywords:** socioeconomic position, lung cancer, stage, population-based, registries

## Abstract

**Introduction:**

We investigated the association between socioeconomic position, stage at diagnosis, and length of period between referral and diagnosis in a nationwide cohort of lung cancer patients.

**Methods:**

Through the Danish Lung Cancer Register, we identified 18 103 persons diagnosed with lung cancer (small cell and non-small cell) in Denmark, 2001–2008, and obtained information on socioeconomic position and comorbidity from nationwide administrative registries. The odds ratio (OR) for a diagnosis of advanced-stage lung cancer (stages IIIB–IV) and for a diagnosis >28 days after referral were analysed by multivariate logistic regression models.

**Results:**

The adjusted OR for advanced-stage lung cancer was reduced among persons with higher education (OR, 0.92; 95% confidence interval (CI), 0.84–0.99), was increased in persons living alone (OR, 1.06; 95% CI, 1.01–1.13) and decreased stepwise with increasing comorbidity. Higher education was associated with a reduced OR for >28 days between referral and diagnosis as was high income in early-stage patients. Male gender, age and severe comorbidity were associated with increased ORs in advanced-stage patients.

**Interpretation:**

Differences by socioeconomic position in stage at diagnosis and in the period between referral and diagnosis indicate that vulnerable patients presenting with lung cancer symptoms require special attention.

Survival after lung cancer remains low in Denmark and is lower than in other western and northern European countries (Berrino *et al*, 2007). The incidence rate of lung cancer is generally strongly associated with socioeconomic position, largely due to differences in smoking patterns (Menvielle *et al*, 2009; Sidorchuk *et al*, 2009). A nationwide Danish cohort study recently demonstrated a difference by socioeconomic position in short-term survival after lung cancer; for instance, the 1-year relative survival was 28% (95% CI, 27–30%) for men with short and 34% (95% CI, 32–37%) for men with higher education (Dalton *et al*, 2008a). This study did not, however, include information on stage of disease, which is a strong prognostic factor in cancer; social differences in stage at diagnosis might therefore explain the social inequality in survival.

Few studies have evaluated the effect of socioeconomic position on lung cancer stage at diagnosis, and the results have been inconclusive (McCarthy *et al*, 2007; Halpern *et al*, 2008; Berglund *et al*, 2010; Booth *et al*, 2010); further, it has never previously been studied in the Danish setting. The Danish tax-funded health-care system provides free access to general practice, outpatient and hospital care. The general practitioners act as gatekeepers to the rest of the health-care system, and carry out initial diagnostic tests and refer to practicing specialists, hospitals or outpatient clinics as needed.

It may be that affluent lung cancer patients benefit more from lung cancer awareness campaigns, leading to shorter delays in seeking treatment or in diagnosis of the disease, which might result in a different stage distribution by socioeconomic position. Further, the differences in the distribution of smokers and comorbidity by social group might lead to differences in the interpretation of warning symptoms, such as cough and dyspnoea, with a corresponding difference in presentation delay.

In a nationwide, population-based cohort of 18 103 patients with lung cancer diagnosed between 2001 and 2008 in Denmark, we investigated the relationship between socioeconomic position and (1) tumour progression, measured as advanced-stage (stages IIIB–IV) *vs* early-stage (stages I–IIIA) lung cancer at the time of diagnosis and (2) the length of the period between referral and diagnosis. We hypothesised that patients’ overall knowledge, reflecting their ability to interpret symptoms, communicate and access health services, is closely related to their educational status. We, therefore, chose the highest attained educational level as the primary socioeconomic variable.

## Materials and methods

In the files of the Danish Lung Cancer Registry, we identified 25 648 persons born between 1920 and 1982 in whom lung cancer was diagnosed between 2001 and 2008 and who were aged ⩾30 years at the time of diagnosis. The Lung Cancer Registry was established in 2001; estimated registration covers >85% and, since 2003, >90% of all lung cancer cases in Denmark (DLCG and DLCR, 2009; Jakobsen *et al*, 2009). We identified 24 229 persons (95%) in the files of Statistics Denmark 2 years before the year of lung cancer diagnosis in order to retrieve their socioeconomic characteristics; we assumed 2 years’ latency to minimise a possible reverse effect of early symptoms of the disease on socioeconomic position. Additionally, we excluded persons who had no information on histological type (*N*=4198; 17%) or hospital ward (*N*=39).

### Classification of stage

Of the 19 992 persons with NSCLC or SCLC, 16 720 persons (84%) were classified as early or advanced stage; 85 cases of clinical stage 0 were excluded. For the 3187 persons with no recorded clinical stage, we classified 1153 persons who had undergone intended curative surgery and 33 persons who were referred to oncological treatment for stage I–IIIA diseases as early-stage lung cancer and 197 persons referred to oncological treatment for stage IIIB–IV diseases as advanced-stage lung cancer. Of the 18 103 persons thus eligible for analysis, 7177 (40%) had a diagnosis of early-stage lung cancer and 10 926 (60%) advanced-stage lung cancer.

### Waiting time

In Denmark, the National Cancer Plan defines the preferable delay between referral and diagnosis of lung cancer as <28 days (National Board of Health, 2010). Waiting time was calculated from the date of initial referral (from general practitioners, private specialists or other hospital wards) to the date at which clinical stage was registered (date of diagnosis). Among the 16 720 patients with a registered stage, 7 had no date of diagnosis and thus the analysis using waiting time as outcome was performed on a data set restricted to 16 713 patients with both a registered stage and date of diagnosis.

### Socioeconomic factors

Information on socioeconomic position was obtained for each lung cancer patient from the Integrated Database for Labor Market Research, which contains annually updated data since 1980 and is run by Eurostat/Statistics Denmark (1995). Education was categorised into short education (i.e., mandatory education of up to 7 and 9 years for patients born before and after 1 January 1958, respectively), medium education (between 8–10 and 12 years, the latest grades of primary school, secondary school, and vocational education) and higher education (>12 years). Disposable income was calculated from household income after taxation and interest per person, adjusted for the number of persons in the household and for the 2000 value of the Danish crown, according to a formula from the Danish Ministry of Finance. We grouped disposable income into low (first quartile), medium (second and third quartiles), and high (fourth quartile). Affiliation to the work market was categorised into working, unemployed, early retirement pension (formerly known as disability pension, which is granted if a person is unable to work permanently due to mental or physical disability and if the disability reduces the ability to work by at least 50%) and age pension (anticipatory pension available from age 60 years and age pension from 65 years). Cohabitation status was defined as living with a partner, irrespective of marital status, or single.

### Comorbid disorders

By linking the personal identification numbers to the files of the Danish National Patient Register, we obtained full histories of diseases leading to hospitalisation from 1978 and, from 1995, outpatient visits for each cohort member to 1 year before the diagnosis of lung cancer (Andersen *et al*, 1999). Based on information on hospital contacts, including dates of discharge and diagnoses coded according to Danish modified versions of ICD-8 and, from 1994, ICD-10, we defined the Charlson comorbidity index, grouped on the basis of the cumulated sum of scores of 0, 1, 2, and ⩾3 (Charlson *et al*, 1987; Dalton *et al*, 2008b).

### Statistical analyses

Logistic regression models were used to examine the simultaneous influence of all socioeconomic and demographic factors of interest on the likelihood of receiving a diagnosis of advanced-stage lung cancer with the GENMOD procedure of SAS 9.1 (SAS Institute Inc., Cary, NC, USA). To account for possible clustering within hospital wards, we used generalised estimating equations with the exchangeable working correlation structure and robust variance estimates.

A three-step model was used. In the first model, each socioeconomic or comorbidity variable was entered alone and adjusted for age and gender. In the second models, the individual exposure variables were additionally adjusted for variables further upstream in the causal pathway (education, cohabiting status, and income). In the final models, analyses were adjusted for age, gender, education, cohabitation status, income, and comorbidity. As there were only minor differences in the estimates obtained with the three models, only data from the first and the final models are shown. In order to explore the influence of affiliation to the working market on the association between socioeconomic position and advanced-stage lung cancer, we ran the logistic regression analyses separately for patients aged <65 years, including work market affiliation in the models.

Tests for interaction (effect modification) between covariates were performed with the Wald test statistic. Investigations of interactions between education and gender, comorbidity, and age, respectively, as well as between comorbidity and sex, and age, respectively, were performed. For the group of patients <65 years, we also tested for an interaction between affiliation to the work market and gender.

For the analysis of waiting time, we investigated the likelihood of a diagnosis >28 days after initial referral in logistic regression models. We used the same three-step model described above, also including stage (advanced or early) as a variable. The analyses were separated by early and advanced stage. Again, as minor differences in estimates were observed between models, only the results of the first (adjustment for age and gender) and the final model are shown.

## Results

### Socioeconomic position and stage of lung cancer

Table 1 gives the descriptive and diagnostic characteristics of the 18 103 lung cancer patients, overall and by educational level. More men than women were diagnosed with lung cancer among persons with medium or higher education whereas the proportions of patients with low income or who were retired or single were higher among those with short education. There were no substantial differences in stage, histological type, or median waiting time by educational group (Table 1).

In general, there were only very slight differences in risk estimates between the age and gender-adjusted and the mutually adjusted analyses (Table 2). No interactions were observed. The odds ratio (OR) adjusted for age, gender, education, income, cohabitation status, and comorbidity for a diagnosis of advanced-stage lung cancer was reduced by 8% for persons with higher education (OR, 0.92; 95% CI, 0.84–0.99) and increased by 6% for persons living alone (OR, 1.06; 95% CI, 1.01–1.13; Table 2). There was a slightly reduced OR of borderline significance for a diagnosis of advanced-stage lung cancer of 0.98 (95% CI, 0.97–1.00) per 5 years increment in age. Having comorbid disorders reduced the OR to 0.88 (95% CI, 0.83–0.92) in persons with a Charlson comorbidity score of 1, to 0.84 (95% CI, 0.77–0.92) in persons with a score of 2 and to 0.73 (95% CI, 0.65–0.81) in those with a score ⩾3 when compared with persons with no comorbidity (Charlson comorbidity score 0; *P*-value for trend <0.001).

For the 7053 patients under 64 years of age, a statistically significant interaction between work market affiliation and gender was observed (*P*=0.01) and models were separated by gender. Similar associations were found between age, education, cohabitation status, and comorbidity and advanced-stage lung cancer (data not shown). In comparison with working men, increased (although of borderline significance) adjusted ORs were found for men who were unemployed (OR, 1.20; 95% CI, 1.00–1.44) or who had retired early because of ill health (OR, 1.18; 95% CI, 0.96–1.44). Unemployed women had a non-significantly reduced OR (0.90; 95% CI, 0.72–1.12), and women who had retired early had a reduced OR (0.80; 95% CI, 0.69–0.92) in comparison with working women.

Separate analyses of the data set after exclusion of the 1386 patients classified as having early- or advanced-stage lung cancer solely on the basis on referral to surgery or oncological treatment gave similar results to the overall analyses (data not shown).

### Socioeconomic position and waiting time

For the analysis of socioeconomic position and waiting time, tests for interactions revealed a significant interaction between stage and age (*P*<0.001) and the analyses were separated by early and advanced stage. The OR adjusted for age, gender, education, income, cohabitation status, and comorbidity for a diagnosis >28 days after referral to hospital increased with age (OR, 1.03; 95% CI, 1.01–1.06 per 5 years) and was higher in men with advanced-stage cancer than in women (OR, 1.11; 95% CI, 1.03–1.21), whereas age and gender did not affect the OR for persons with early-stage lung cancer (Table 3). Higher education was associated with a reduced OR for a diagnosis >28 days after referral among patients with both early- and advanced-stage cancer (OR, 0.82; 95% CI, 0.70–0.96 and OR, 0.82; 95% CI, 0.72–0.93, respectively), as was medium education among early-stage patients (OR, 0.87; 95% CI, 0.81–0.94) with *P*-values for trend of <0.001 and 0.01 in early- and advanced-stage patients (Table 3). High income was associated with lower ORs for a diagnosis >28 days after referral although failing to reach statistical significance for patients with advanced-stage cancer (Table 3). Male gender and severe comorbidity (Charlson comorbidity scores of 2 or higher) were associated with increased ORs in advanced-stage lung cancer patients (*P*-value for trend <0.001) but not significantly so among patients with early-stage cancer (Table 3).

To check for co-linearity between education and income, all models were run both with and without income and very little change was observed in risk estimates indicating no co-linearity (data not shown). Some 17% of the material was excluded due to missing histology; mutually adjusted regression models revealed that older age, living alone and having comorbidity was significantly associated with the OR for having no histology while there was no association between gender, education, or income and having no histology (Table 4).

## Discussion

In this nationwide population-based study of stage at the time of diagnosis of lung cancer, short education and living alone were associated with higher risks for a diagnosis of more advanced disease. Furthermore, short education was associated with a longer than recommended time period between referral and diagnosis. Longer than recommended periods between referral and diagnosis were found for low income patients with a diagnosis of early-stage lung cancer, and for patients with advanced-stage lung cancer who were male, older and had severe comorbidity.

A recent population-based study in mid-Sweden of 3370 patients with NSCLC diagnosed in 1996–2004 showed no association between education and stage at diagnosis (Berglund *et al*, 2010). A Canadian study of 12 276 NSCLC patients diagnosed in 2003–2007 showed no difference in stage distribution by quintile of median area-based household income, but this study did not include information on education (Booth *et al*, 2010). We found evidence of an education gradient in stage at diagnosis among Danish patients with either NSCLC or SCLC, both of which were included because of the similarity in symptoms, the diagnostic procedures and the comparability of the staging of these groups of lung cancer; however, exclusion of SCLC from the data set resulted in similar results (data not shown). In line with our findings, a study in the United States of almost 700 000 patients with lung cancer diagnosed in 1998–2004 showed ORs of 1.3 (95% CI, 1.3–1.4) for a diagnosis of stages III–IV rather than stage I for persons insured by Medicaid (for low income or the medically needy) and 2.2 (95% CI, 2.1–2.3) for persons with no health insurance when compared with persons who were privately insured (Halpern *et al*, 2008). The present study is the largest population-based study outside the United States to be published, and our results support the notion that social differentials in stage at diagnosis might contribute to the social inequality in lung cancer survival, as has been demonstrated in countries with different levels of social security and welfare (Rachet *et al*, 2008; Dalton *et al*, 2008a; Berglund *et al*, 2010; Booth *et al*, 2010).

In accordance with some (Osborne *et al*, 2005; McCarthy *et al*, 2007; Frederiksen *et al*, 2008) but not all (Dalton *et al*, 2006; Berglund *et al*, 2010) studies of social position and stage of cancer, we found that living alone was associated with higher odds for late diagnosis of lung cancer than if living in a relationship. Living with a partner might reduce the delay in seeking medical help after symptoms are experienced and might help in navigating the diagnostic pathway, which includes several sectors of the health system. Furthermore, higher smoking prevalence and lower cessation rates have been observed among persons living alone (Broms *et al*, 2004; Osler *et al*, 2008; Giordano and Lindstrom, 2010).

The factor most strongly associated with stage in the fully adjusted analyses was comorbidity, which was associated with lower ORs for advanced-stage disease. This is plausible from a clinical point of view, because persons with chronic conditions are more likely to require frequent, periodic medical care, resulting in closer clinical monitoring than healthier persons. As a substantial proportion of lung cancer patients have physical disabilities that compromise their lung function, they may have more frequent X-rays or CT scans, which could detect early lung cancers.

The finding that comorbid disorders might lower the odds for late-stage disease at diagnosis is in line with the findings of a study in the United States based on SEER data, of 4626 persons with social security disability insurance entitlement to Medicare, who received a diagnosis of NSCLC, in which the adjusted OR for stages III–IV was 0.76 (95% CI, 0.72–0.81) in comparison with people without social security disability insurance (McCarthy *et al*, 2007).

We observed a difference by gender among lung cancer patients of working age. Comorbidity overall was associated with a lower OR for a diagnosis of advanced-stage lung cancer, but men who were unemployed or early retirement pensioners (many of whom have a Charlson comorbidity score, as early retirement can be granted in Denmark only if working ability is permanently reduced by >50%) were at increased odds for a diagnosis of advanced-stage lung cancer, whereas unemployed women or female early retirees were not. This finding indicates a vulnerable group of men with severe chronic comorbidity (Dalton *et al*, 2008b), who might not receive as much surveillance as their female counterparts or other persons with comorbid conditions who are working. A similar finding has to our knowledge not been reported earlier and might be a chance finding; however, if it can be replicated in further studies, the identification of a complex association between comorbidity, gender and lung cancer stage adds valuable information to our understanding of how socioeconomic position influences health outcomes.

We also observed that level of education is associated with time between referral and diagnosis of either early- or advanced-stage lung cancer. A similar result was obtained in the Swedish study of early-stage NSCLC patients, with a difference in median waiting time of 32 and 17 days, respectively, for patients with low and high education (Berglund *et al*, 2010). As the authors found no difference by education in stage at diagnosis, however, they concluded that their finding was of no clinical significance. Few other studies have explored the association between socioeconomic position and delay. A British study found that age and marital status were associated with longer overall diagnostic delay in lung cancer, but that age, gender, marital status, or social or ethnic group did not influence the delay to referral or secondary care (Neal and Allgar, 2005), which would encompass the period between referral and diagnosis that we investigated. We were unable to investigate how socioeconomic position influences the delay between symptom debut and contact with a doctor or the delay between first contact with a doctor and referral; however, our finding of a difference by education and to some degree income in the length of the diagnostic process and the stage at time of diagnosis – in a country with equal, free access to the health-care system – draws attention to practices of care in both referral and the diagnostic work-up of lung cancer.

The strengths of this study include the availability of high-quality clinical information on a population basis, from a clinical database with national coverage of about 90% of lung cancers diagnosed in the period. The detailed information in the clinical database enabled us to investigate the early disease trajectory, as we were able to retrieve information on the interval between referral and diagnosis as well as on clinical stage. The range of information in the database enabled us to infer clinical stage from referral to treatment for almost half of the 15% of patients for whom a primary clinical stage was not reported, thus increasing the external validity of the study. We excluded a substantial part of the patient group (17%) due to lack of information on histology. However, our finding that factors like age, cohabitation status, and comorbidity were associated with missing information on histology indicates that the strength of the associations observed between these factors and stage or waiting time might be underestimated. Linkage with other administrative registries with information collected for purposes independent of the study hypotheses and covering the entire Danish population ensured minimal selection and information bias, whereas the availability of individual-level socioeconomic position indicators reduced the likelihood of misclassification of exposure, which could arise if area-based socioeconomic position measures were used (Galobardes *et al*, 2006).

The inclusion of information on comorbidity from the Charlson comorbidity index, which is a validated instrument (Charlson *et al*, 1987) is clearly another strength of the study. It is, however, not possible to distinguish between the mildest and the most severe cases in the categories of diseases, because the index is based on discharge diagnoses from inpatient or outpatient admissions only. Furthermore, patients who were treated for their disease solely by their general practitioner score 0 in this index, which could lead to misclassification of exposure and residual confounding when comorbidity is treated as exposure or confounder. We were furthermore unable to explore the mechanisms underlying the association between short education, living alone, older age and advanced stage of lung cancer at diagnosis as we had no information on the time of symptom onset, first visit to the general practitioner or delay before diagnostic procedures. Finally, we were unable to adjust for smoking status. There may be an educational gradient among people who have stopped smoking (Osler *et al*, 2001; Pisinger *et al*, 2008). Among former smokers one would expect increased awareness, if symptoms as cough or dyspnoea arise due to a developing lung cancer as these symptoms might be interpreted as smoking related among smokers. An educational gradient in smoking cessation might therefore lead to differential awareness of symptoms by education related to both smoking and lung cancer, and thus possibly have a role in an educational gradient in stage at diagnosis of lung cancer.

In spite of the insufficient evidence of a positive association between short diagnostic delay, early stage and prognosis of lung cancer, it is reasonable to assume that better survival rates can be achieved when lung cancer is diagnosed at an early stage, given the potential for curative treatment. The strengths of the associations we observed on stage at diagnosis and waiting time suggest that compared with the effect of socioeconomic position on lung cancer incidence the social gradient on these end points is moderate. Still, our results indicate that the pathway from referral due to a suspicion of cancer to diagnosis differs by socioeconomic position for lung cancer patients in Denmark. The finding that patients with short education, low income or who live alone have a higher risk for a longer period than recommended between referral and diagnosis and for advanced-stage lung cancer calls for greater attention to these groups of patients when they enter the health-care system with symptoms indicative of lung cancer.

Implications of such findings could be that optimised diagnostic processes securing early referral and navigation of vulnerable patients through different sectors of the health system should be offered to groups defined by low socioeconomic position or who live alone.

## Figures and Tables

**Table 1:** Descriptive characteristics of 18 103 persons born 1920–1978 in whom non-small cell or small cell lung cancer was diagnosed after the age of 30 years, Denmark, 2001–2008, by educational level

	**Short education (*N*=7643)**		**Higher education (*N*=2028)**		**Higher education (*N*=2028)**		**Total (*N*=18 103)**	
**Characteristic**	***n***	**%**	***n***	**%**	***n***	**%**	***n***	**%**
*Period*								
2001–2002	1554	20	1583	19	360	18	3497	19
2003–2004	2058	27	2148	25	520	26	4726	26
2005–2006	2030	27	2286	27	553	27	4869	27
2007–2008	2001	26	2415	29	595	29	5011	28
								
*Age (years)*								
30–54	315	4	1439	17	313	15	2067	11
55–64	1778	23	2608	31	600	30	4986	28
65–74	3389	44	2909	35	708	35	7006	39
⩾75	2161	28	1476	18	407	20	4044	22
								
*Gender*								
Male	3725	49	4858	58	1280	63	9863	54
Female	3918	51	3574	42	748	37	8240	46
								
*Disposable income*								
Low	3393	44	2316	27	297	15	6006	33
Medium	3751	49	4511	54	955	47	9217	51
High	499	7	1605	19	776	38	2880	16
								
*Affiliation to work market*								
Working	1280	17	3050	36	916	45	5246	29
Unemployed or other	330	4	613	7	99	5	1042	6
Early retirement	1278	17	984	12	124	6	2386	13
Pensioner	4299	56	3497	41	809	40	8605	48
Unknown	456	6	288	3	80	4	824	5
								
*Cohabitation status*								
Living with someone	4841	63	5891	70	1475	73	12 207	67
Living alone	2802	37	2541	30	553	27	5896	33
								
*Charlson comorbidity index score*								
0	3952	52	4806	57	1234	61	9992	55
1	1668	22	1722	20	373	18	3763	21
2	1068	14	1075	13	225	11	2368	13
⩾3	955	13	829	10	196	10	1980	11
								
*Stage*								
Low (I–IIIA)	3061	40	3271	39	845	42	7177	40
High (IIIB–IV)	4582	60	5161	61	1183	58	10 926	60
								
*Primary staging*								
Yes	7108	93	7771	92	1841	91	16 720	92
No	535	7	661	8	187	9	1383	8
								
*Histological type*								
Non-small cell	6619	87	7403	88	1794	88	15 816	87
Small cell	1024	13	1029	12	234	12	2287	13
								
*Median waiting time (days (max))*								
Overall	21 (482)		20 (2573)		20 (384)		20 (2573)	
97.5% of sample (⩽82 days’ delay)	20		20		20		20	
95% of sample (⩽63 days’ delay)	20		20		20		20	
No information	539	7	664	8	187	9	1390	8

**Table 2:** Age and gender adjusted and multivariate adjusted odds ratios (ORs) with corresponding 95% confidence intervals for advanced-stage non-small or small cell lung cancer (IIIB–IV) in 18 103 persons aged ⩾30 years at time of diagnosis, Denmark, 2001–2008

		**Age and gender adjusted analyses**		**Mutually adjusted analyses** a	
**Variable**	***N* (late stage)/*N* (total)**	**OR**	**95% confidence interval**	**OR**	**95% confidence interval**
Age per 5 years		0.97	0.96–0.99	0.98	0.97–1.00
					
*Gender*					
Male	5877/9863	0.95	0.91–0.99	0.97	0.92–1.02
Female	5049/8240	1		1	
					
*Educational level*					
Short	4582/7643	1		1	
Medium	5161/8432	0.99	0.93–1.06	0.99	0.93–1.06
Higher	1183/2028	0.92	0.84–1.01	0.92	0.84–0.99
					*P-value for trend 0.08*
					
*Disposable income*					
Low	3564/6006	1		1	
Medium	5623/9217	1.03	0.97–1.10	1.05	0.98–1.12
High	1739/2880	0.97	0.88–1.08	0.99	0.89–1.11
					*P-value for trend 0.81*
					
*Cohabitation status*					
Living with partner	3665/5896	1		1	
Single	7261/12 207	1.06	1.00–1.12	1.06	1.01–1.13
					
*Charlson comorbidity index score*					
0	6249/9992	1		1	
1	2222/3763	0.88	0.84–0.93	0.88	0.83–0.92
2	1371/2368	0.84	0.77–0.92	0.84	0.77–0.92
⩾3	1084/1980	0.73	0.66–0.82	0.73	0.65–0.81
					*P-value for trend <0.001*

a ORs are mutually adjusted as well as adjusted for hospital ward in generalised estimating equations.

**Table 3:** Age and gender adjusted and multivariate adjusted odds ratios (ORs) with corresponding 95% confidence intervals (CIs) for a diagnosis >28 days after referral, among 16 713 persons with non-small cell or small cell lung cancer aged ⩾30 years, Denmark, 2001–2008, by clinical stage

	**Stage I–IIIa**			**Stage IIIB–IV**		
	**OR (95% CI)**			**OR (95% CI)**		
**Variable**	***N* (>28days)/ *N* (total)**	**Age and gender adjusted**	**Mutually adjusted** a	***N* >28days)/ *N* (total)**	**Age and gender adjusted**	**Mutually adjusted** a
Age per 5 years		1.00 (0.97–1.04)	0.98 (0.95–1.01)		1.05 (1.03–1.07)	1.03 (1.01–1.06)
						
*Gender*						
Male	1529/3344	1.02 (0.90–1.14)	1.03 (0.91–1.16)	1503/5767	1.11 (1.02–1.21)	1.11 (1.03–1.21)
Female	1203/2648	1	1	1193/4954	1	1
						
*Educational level*						
Short	1234/2597	1	1	1169/4507	1	1
Medium	1195/2710	0.86 (0.80–0.93)	0.87 (0.81–0.94)	1266/5058	0.96 (0.88–1.04)	0.97 (0.89–1.05)
Higher	303/685	0.79 (0.69–0.91)	0.82 (0.70–0.96)	261/1156	0.80 (0.70–0.91)	0.82 (0.72–0.93)
			*P-value for trend* <0.001			*P-value for trend* 0.01
*Disposable income*						
Low	960/2076	1	1	884/3502	1	1
Medium	1370/3007	0.93 (0.83–1.03)	0.94 (0.84–1.06)	1410/5520	1.01 (0.91–1.11)	1.02 (0.92–1.12)
High	402/909	0.80 (0.69–0.91)	0.85 (0.74–0.98)	402/1699	0.88 (0.79–0.99)	0.93 (0.83–1.03)
			*P-value for trend* 0.06			*P-value for trend* 0.20
						
*Cohabitation status*						
Single	859/1877	1.05 (0.96–1.14)	1.02 (0.93–1.11)	877/3601	0.99 (0.92–1.07)	0.98 (0.90–1.07)
Living with partner	1873/4115	1	1	1819/7120	1	1
						
*Charlson comorbidity index score*						
0	1378/3093	1	1	1474/6142	1	1
1	607/1311	1.06 (0.94–1.18)	1.05 (0.93–1.18)	546/2187	1.01 (0.92–1.11)	1.00 (0.91–1.10)
2	383/826	1.07 (0.94–1.23)	1.07(0.94–1.22)	384/1337	1.22 (1.11–1.34)	1.21 (1.10–1.33)
⩾3	364/762	1.14 (0.98–1.33)	1.11 (0.95–1.30)	292/1055	1.18 (1.04–1.33)	1.17 (1.03–1.33)
			*P-value for trend* 0.09			*P-value for trend* <0.001

a ORs are mutually adjusted as well as adjusted for hospital ward by generalised estimating equations.

**Table 4:** Age and gender adjusted and mutually adjusted odds ratios (ORs) with corresponding 95% confidence intervals for having missing histology in 24 229 patients with lung cancer, Denmark, 2001–2008

		**Age and gender adjusted analyses**		**Mutually adjusted analyses**	
**Variable**	***N* (missing)/ *N* (total)**	**OR**	**95% confidence interval**	**OR** a	**95% confidence interval**
Age per 5 years		1.11	1.09–1.13	1.11	1.09–1.13
					
*Gender*					
Male	2248/13 086	0.95	0.89–1.02	0.97	0.91–1.04
Female	1950/11 143	1		1	
					
*Educational level*					
Short	1812/10 241	1		1	
Medium	1919/11 247	0.96	0.89–1.03	1.07	0.99–1.15
Higher	467/2741	0.96	0.85–1.07	1.05	0.93–1.18
					
*Disposable income*					
Low	1505/8105	1		1	
Medium	2069/12 311	0.89	0.82–0.95	0.95	0.88–1.03
High	624/3813	0.86	0.77–0.95	1.05	0.94–1.18
					
*Cohabitation status*					
Living with partner	2654/16 184	1		1	
Single	1544/8045	1.21	1.13–1.29	1.17	1.09–1.26
					
*Charlson comorbidity index score*					
0	2108/13 130	1		1	
1	926/5051	1.17	1.08–1.28	1.11	1.01–1.21
2	613/3259	1.21	1.10–1.34	1.12	1.01–1.24
⩾3	551/2789	1.29	1.16–1.43	1.16	1.04–1.29

a ORs are mutually adjusted as well as adjusted for hospital ward by generalised estimating equations.
